# What influences individuals to invest in improved sanitation services and hygiene behaviours in a small town? A formative research study in Babati, Tanzania

**DOI:** 10.1371/journal.pone.0270688

**Published:** 2022-07-21

**Authors:** Gabriel Malima, Hoyce Mshida, Revocatus Machunda, Francis Moyo, Joseph Banzi, Om Prasad Gautam, Mbaye Mbeguere, Kyla Smith, Sandy Cairncross, Karoli N. Njau

**Affiliations:** 1 Nelson Mandela African Institution of Science and Technology, Arusha, Tanzania; 2 WaterAid Tanzania, Dar es Salaam, Tanzania; 3 WaterAid UK, London, United Kingdom; 4 London School of Hygiene and Tropical Medicine, London, United Kingdom; Universiti Malaysia Terengganu, MALAYSIA

## Abstract

Sub-Sahara African countries face immense challenges in ensuring adequate sanitation and hygiene behaviours to the rapidly growing populations. Attempts to address these challenges require empirical evidence to inform policy and planning. We contribute toward that goal by unveiling findings of formative research conducted in Babati, a rapidly growing town in Tanzania. We conducted a cross-sectional study involving 486 households, to unwind motives and barriers for individuals to invest in improved sanitation services and hygiene behaviour change. We used several methods including household survey, focus group discussions, behaviour observations and spot checks. The findings revealed that households derive their motivation to invest in improved sanitation and hygiene practices from comfort, raising social status, and the need for personal safety and privacy. Other motives include fear of penalties and fines and fear of disease outbreaks, whilst the barriers include, limited water availability and accessibility, environmental factors, property rights, cultural issues, financial constraints, and a person’s attitude. Quantitative data were subjected to multivariate analysis to identify determinants of households to invest in sanitation and hygiene practices. The logistic regression analyses revealed that sources of water, property rights, and education level were the main determinants of households to invest in sanitation and hygiene facilities, while household income was the main determinant for households to invest in both construction of handwashing facility and water treatment. We argue that the initiative to promote sanitation and hygiene behaviour change in small towns should focus on promoting motivation factors and abating the determinant factors identified in this study.

## 1. Introduction

Reports by the United Nations Children’s Fund (UNICEF) and the World Health Organization (WHO) show that in the year 2020, about 3.6 billion people around the world had no access to safely managed sanitation, and of these, 494 million people were practising open defecation [1]. The report by UNICEF also shows that worldwide 2.3 billion people lack basic handwashing facilities with water and soap at homes (2020), and 57% of schools lack handwashing facilities with water and soap in 2019 [2]. Tanzania is one of the sub-Sahara African countries that face many sanitation and hygiene challenges. Only 55% and 15.4% of the country’s population has access to safe water and sanitation, respectively [3]. Poor sanitation and hygiene in Tanzania is a leading cause of premature deaths and waterborne diseases [4]. Poor sanitation costs Tanzania approximately US $206 million per year in costs linked to healthcare, premature death, sickness and losses in productivity and time [4]. The nature of sanitation challenges in Tanzania and sub-Saharan Africa requires a collective effort of institutions and individual households to improve sanitation services. Hygiene challenges also require a collective effort and commitment of both institutions and individuals. Hygiene practices rely on both individual motivations and behaviours, and the availability of an enabling environment such as sufficient water supply, sanitation services and hygiene infrastructure and products with cues, and nudges to remind and reinforce individuals to use them. This necessitates the need to build empirical evidence on what drives or blocks households/individuals from investing in improved sanitation and hygiene to effectively inform policy-makers and intervention programs.

Previous studies in Tanzania have identified several determinants to access improved sanitation and hygiene facilities. The evaluation of the Tanzania’s National Sanitation Campaign in 26 regions of Tanzania mainland revealed that the key determinants for access to improved sanitation and hygiene services were economic status of the households, education level of the head of household and geographical location of the household [5]. A study on access to water and sanitation facilities among the pastoralists in Northern Tanzania by Nyanza et al [6] identified other determinants to access a sanitation facility at a household including family size, presence of under-five years of age in the household, history of diarrhoeal diseases, having ever received education on sanitation and motivation for improvement in defecation places. Presence of sandy soils that hamper digging standard pits and inability of households to construct latrines, were also reported as key barriers towards construction of improved latrines in Kahama, Tanzania by Kamara et al [3].

Regarding access to hygiene services, a study conducted in Dar es salaam by Pickering et al [7] revealed that a mother’s educational attainment, use of an improved toilet, an infant in the household, and dissatisfaction with the quantity of water available for hygiene, were key household characteristics associated with hand fecal contamination. Handwashing at critical times is crucial to deal with fecal contamination and improve hygiene. An assessment of availability of handwashing facilities in households from four East African countries (Tanzania, Kenya, Uganda and Rwanda) revealed that the type of residence, household wealth, number of children, age, sex and education of the household head were strong predictors of having a handwashing facility in the household [8]. A sanitation study in school settings by Okello et al [9] in Kagera region in Tanzania found that motivation for handwashing practices was enhanced by emotional drivers such as disgust, fear and nurture as well as newly established handwashing stations.

While Tanzania has achieved significant economic growth over the past decade, with a modest reduction in poverty, the country still lags behind in expanding and sustaining basic water, sanitation and hygiene (WASH) coverage [10]. This situation is further affected by the *rapidly growing population* especially in small fast-growing towns. Thus, information about the current state of sanitation and hygiene is highly needed to inform evidence-based strategies to address WASH challenges in small towns. Identification of the factors that influence individuals to invest in sanitation and hygiene would help efforts to address the current state in Tanzania and other countries with similar characteristics. In this line, we conducted a study to shed light on the factors affecting individuals to invest in improved sanitation services and hygiene behaviours in small towns.

## 2. Material and methods

The study area for this research was Babati Town, a fast-growing, small town in Tanzania. with a population of about 110,000 individuals [11]. The town sits at a crossroads between Arusha, Singida, Dodoma, Mbulu and Simanjiro. The recent (2016) completion of a paved road passing right through Babati town from Dodoma on route to Arusha (two of Tanzania’s largest cities), is one of the key reasons behind Babati’s expansion. Additionally, the Region of Manyara, in which it belongs, recorded the highest growth rate of 3.2% (between 2002–2012), making it the region with the third highest growth rate in Tanzania, after Dar es salaam (5.6%) and Mjini Magharibi (4.6%) [12]. Economic activities of Babati are primarily agrarian which attract people to the area, for example, presence of several large sunflower processing plants, and many small processing plants for various agricultural products, attract migrants from the rural areas [13].

Babati’s urban settlements are surrounded by rural communities characterized by low population density and small settlements. The town has eight wards, of which two are urban wards and six are peri-urban wards. The data collection exercise was conducted in all wards from November 2016 to June 2017. We employed a cross-sectional study design to collect both qualitative and quantitative data. Data was collected through a household survey, spot check, Focus Group Discussions (FGDs) and Motives and Barriers analyses. We also conducted FGDs with students in primary schools, secondary schools and colleges (Table 1).

**Table 1:** Data collection techniques, sample size and tools.

Methods	Sample size	Tools
Household survey	486 households	Questionnaire
Focus group discussion	33 sessions in 9 schools and 2 colleges (396 respondents); 24 sessions in communities (168 household adult members)	FGD checklist
Motive and barrier exercise	8 sessions (80 household adult members)	Motives and barrier mapping checklist
Spot-check	486 households; 31 schools; 2 vocational training colleges	Spot-check checklist

For the household survey, a multi-stage stratified sampling method was employed. The first stage involved dividing the study area into wards. In the second stage, the wards were divided into blocks in which the villages (for the peri-urban wards) and streets (for the urban wards) were used as blocks. The blocks were randomly selected to remain with four blocks from each ward. Local government officials provided the lists of households of each of the selected block for sampling, and simple random sampling method was used to select actual households for the survey. The household survey was done by using a questionnaire designed to elicit information concerning socio demographic information, knowledge, reported and observed practices and social norms link to each of the targeted behaviours, modified from the existing validated UNICEF—Knowledge, Attitude and Practice (KAP) tool [14–16]. This questionnaire was then translated to Kiswahili, a language spoken by almost all Tanzanians. We trained enumerators and pre-tested data collection tools for three days in a nearby semi-urban township with similar socio-economic settings to Babati town. During the actual household survey, enumerators exchanged filled questionnaires to allow their colleagues to check if the answers provided were valid and complete. The questionnaires were also checked by supervisors in the field. Additional quantitative data were collected through spot-check during household surveys, where enumerators visited the latrines, handwashing facilities and filled the spot-check checklist. The spot-checks were also conducted in schools and colleges. The observations were recorded via checklist and notes. The checklist included types of latrines, a state of cleanness of the latrine and availability of a handwashing station with soap and water. Regarding cleanness measurement, a latrine was recorded to be dirty if human excreta and/or worms were seen on the floor/drop hole or a wall, and clean if none of them was observed.

Regarding FGDs, a total of 24 FGDs were conducted in eight wards involving adult members from selected households. FGDs regarding menstrual hygiene management (MHM) were conducted with female participants only, while other FGDs sessions comprised both male and female attendees. Three topics were discussed; sanitation and hygiene status (8 mixed FGDs), willingness to pay for sanitation (8 mixed FGDs) and hygiene services and menstrual hygiene management (8 female-only FGDs). A total of 33 FGDs sessions were also conducted in nine schools and two colleges. Of these, 11 male-only FGDs and 11 female-only FGDs were conducted to assess school WASH status in schools. The other FGDs conducted assessed the MHM (11 female-only FGDs). The participants for the MHM FGD were purposively selected among the girls at menarche. The checklist for the MHM FGD was adopted from UNICEF publication [17]. The school FGDs involved primary school students from three public schools, secondary school students from three public schools and two private schools and college students from two public colleges.

Motives and Barriers exercises were used to identify the motivational factors and barriers to construct an improved latrine and practice hygiene behaviour. The exercises were conducted in all wards of Babati town (one session in each ward). Each session involved 6 to 12 male and female participants. The Motive and Barrier exercise guide used was designed following the WaterAid guideline [18].

The FGDs and Motives and Barriers exercises were conducted according to the protocol developed by the research team based on the Behaviour Centred Design (BCD) approach [19]. The protocol was designed to help organize and conduct the FGDs and motive and barriers exercises. It provided information on the recruitment of FGD participants and the management of the FGD sessions and Motive and Barrier exercises. Following the protocol, all the FGD checklists and the motive and barrier exercise guides were translated from English to Kiswahili then pre-tested in a nearby semi-urban area, outside of Babati Town. All FGDs and motives and barriers exercises were recorded using a voice recorder, after obtaining respondent informed consent. For the case of primary school and secondary school students who most of them were below 18 years old, consent to participate in the FGDs was obtained from students’ parents/guardians. School teachers were asked to inform the children’s parents the objective of the research and seek their consent on the behalf of the research team.

The data saturation point was determined when the facilitator began to hear the same comments again and again. At that point, probing stopped because the new data tend to be redundant of data already collected.

Before the start of data collection, we obtained ethical clearance with Serial number NIMR/HQ/R.8a/Vol.IX/2335 from the National Institute for Medical Research (NIMR), Tanzania. Additionally, informed consents were signed by all study participants before questionnaire filling.

## 3. Data analysis

### 3.1. Qualitative data

Audio records from FGDs and Motives and Barriers exercise were transcribed, and the transcripts were then translated from Kiswahili to English. Thematic analysis was used to group the data accordingly to similarities through development of codes and themes. A computer software, NVivo (Version 11) was used to assist in coding and theme development. The transcripts with unstructured texts were imported into the software, then the relevant passages in the transcripts were coded. Generated coded information was then summarized according to main themes, sub-themes and codes (Tables 2 and 3).

**Table 2:** Measures for investing in sanitation.

Theme	Sub-theme	Codes
Theme 1: Motives to invest in improved sanitation practices	Institutional aspects	• existing bylaws that fine the households with no latrines education to create awareness on the consequences of poor sanitation practices (*NGOs*, *government health workers)*
	Safety	• fear of contracting a disease (e.g. cholera outbreak) • the need to have a safe place for defecation/urination
	Social status /influence	• having a latrine raises social status • symbolic status linked with having a latrine
	Nurture	• parents construct improved latrines to prevent children from falling into pit latrines that have no slab
	Comfort	• Comfort; it is easier to use the latrine for defecation than using bushes, valleys or go to distant places for defecation • feel clean when using latrine to defecate/urinate
Theme 2: barriers to invest in improved sanitation practices	Institutional aspects	• weak enforcement of the sanitation by-laws in some villages and streets in town
	Poor soil condition	• poor soil condition that makes pit side walls collapse easily/weak • high water table and rocky soils make it difficult to construct durable pits/septic tanks
	Land availability	• availability of land to dig new pit latrine whenever the old one is full
	Limited water availability	• limited water availability preventing installation of flush or pour-flush toilets • household not connected to water supply cannot use flush toilets
	Financial constraints	• cost of constructing improved latrines is not affordable • household’s income is low • sludge emptying services are expensive
	Culture	• Tradition that prohibits a man from sharing a latrine with his in-laws

**Table 3:** Measures for investing in hygiene.

Theme	Sub-theme	Codes
Theme 1: Motives to wash hands with water and soap	Feel of disgust	• feeling of disgust (washing hands to simply remove dirt/dust) • wash hands to look clean and walk around with clean hands
	Comfort	• wash hands to feel good; fresh and clean • wash hands where a handwashing station is close to the latrine
	Safety	• fear of contracting a disease (diarrhoea/stomach related diseases)
Theme 2: Barriers to wash hands with water and soap	Institutional aspects	• lack of by-laws that force people to construct handwashing facilities • person’s attitude
	Culture	• religious beliefs (e.g. “God offers protection”)
	Financial constraints	• lack of money to buy soap often • limited resources to construct handwashing facilities • household members of the households that buy water from vendors cannot wash hands frequently
Theme 3: Motives to treat water	Safety	• fear of contracting water-borne diseases such as cholera and diarrhoea
Theme 4: Barriers to treat water	Source of water	• a belief that piped water (from the town water authority) is safe • a belief that the water from the spring is not contaminated
	Financial constraints	• household cannot afford to buy fuel to boil drinking water every day • large family size
	Culture	• religious beliefs (e.g. “God offers protection”)
	Taste	• water changes taste after being treated • water smells like smoke after being boiled
Theme 5: Motives to maintain cleanness of the latrine	Institutional aspects	• fear of being fined (approx. 25 USD)
	Social pressure	• social pressure (e.g. feeling of being embarrassed because of having a dirty latrine)
	Disgust	• feeling of disgust (when using a latrine with visible faeces and urine)
	Safety	• fear of contracting diseases (from dirty latrines)
Theme 6: Barriers to maintain cleanness	Water availability	• limited availability of water (water to clean household latrine) • belief that child faeces are less harmful than the adult one
Theme 7: Motives to maintain proper MHM	The use of sanitary pads	• one wants to feel confident • comfortable and less fear of leaking (of menstrual blood).
Theme 8: Barriers to maintain proper Menstrual hygiene management	The use of sanitary pads	• the sanitary pads are not affordable • the sanitary pads are not often available in rural areas • lack of designated places to dispose used sanitary materials allergic reactions after using pads • a fear of getting urinary infections after using pads • lack of changing rooms in schools • limited water availability and lack of soap in the school latrines

### 3.2. Quantitative data

We used a Chi-square test to analyse the association between the type of latrine (as a dependent variable) and predictor variables including household head sex, education level, and marital status, family size, household monthly income, residence ownership, wealth quintile and household’s source of domestic water. Variables that showed significant coefficients in the chi-square tests were subjected to multinomial logistic regression to test the variables influence on the type of latrine owned by the household.

The presence of a handwashing facility was assessed using binary responses (Yes/No), hence this variable was treated as a dichotomous dependent variable. We used a Chi-Square test to determine the association between the presence of a handwashing station and household head sex, education level, and marital status, family size, household monthly income, residence ownership, wealth quintile and household’s source of domestic water. The variables which showed significant coefficients were subjected to binary logistic regression to test their influence on the household decision to own a hand washing station. Water treatment practice was also treated as the dichotomous dependent variable, hence similar statistical tests were used to determine the relationship between water treatment practices and household demographic characteristics.

## 4. Results

### 4.1. Demographic characteristics of the study participants

The majority (60%) of the respondents interviewed had attained primary education. Primary education level is the typical level for many adults in Tanzania [20]. About half of the respondents (57%) mentioned farming as a primary occupation and livestock keeping as the secondary occupation of the head of the household. The mean average monthly income of the household was 101 USD, the minimum was 4 USD, and the maximum income was 1,434 USD. The majority of the respondents (43.4%) reported an average monthly income of less than 50 USD. This average monthly income level is similar in many other regions in Tanzania [21]. The majority of the households (85%) were living in privately owned households, and the number of household members ranges between 4 to 8 members (Table 4).

**Table 4:** Socio-demographic characteristics of study participants.

Socio-demographic characteristics	Frequency	%
**Sex**		
Female	305	63
Male	181	37
**Marital Status**		
Single	48	9.9
Married‎‎/Cohabiting	358	73.7
Divorced‎/Separated	33	6.8
Widow	47	9.7
**Education level**		
No formal education	46	11.6
Primary education	289	73.2
Secondary education and above	60	15.2
**Family size** (members)		
1 to 4	173	35.6
4 to 8	274	56.4
9 and above	39	8
**Head of household**		
Male-headed household	383	78.8
Female-headed household	103	21.2
**Primary occupation of the head of household**		
Farmer (crop grower)	277	57
Businessperson	82	17
Salaried worker	39	8
Casual labourer	24	5
Fishermen	13	3
Livestock Keeper	11	2
Others	40	10
**Household ownership**		
Privately owned house	413	85
Relative house	19	43.9
Rental house	54	11.1
**Wealth quintile**		
First	346	87.2
Second‎‎/Third‎/Fourth	51	12.8
Fifth Quintile	0	0
**Household Income (US$ per month)**		
49 and below	172	43.4
50–99	116	29.3
100–149	48	12.1
150–199	19	4.8
200 and above	41	10.4

### 4.2. Motives and barriers to invest in sanitation

FGDs and the Motives and Barriers analysis identified the following as main factors that motivate or push individual households to invest in sanitation: existing rules and regulations, fear of disease outbreaks (e.g. cholera outbreak), comfort–easy to use, raising social status–symbolic status and awareness on consequences of poor sanitation link with having a latrine, and the need to have a safe place for defecation/urination. Existing rules and regulations were the main reasons for a household to construct a latrine because most feared they would be fined (cause financial loss) by the inspecting officers. The fines for failure to construct a latrine was approx. 25 USD. Safety for children was a key factor for the household to construct improved latrines to prevent children from falling into latrine pits when using the latrines (nurture motive). On the other hand, education and awareness about proper sanitation management and the associated consequences of poor sanitation is needed. For example, training and awareness campaigns provided by Non-Governmental Organization (NGOs) and government health workers were frequently mentioned to have influenced people to adopt improved sanitation facilities.

Environmental factors (such as poor soil condition, high water table and rocky soils) were mentioned as barriers to construct improved pit latrines. During the FGD, one of the respondents said:

“We are not constructing traditional pit latrines because when it rains …… the pit collapses. Even if you construct an improved one, it is nothing, it will collapse anyway due to the nature of the soil here”.

Other barriers mentioned include limited access and availability to water supply and cultural issues/tribal traditions such as the tradition that is practised among some nomadic ethnic groups that prevent a man from sharing a latrine with his in-laws. Regarding the limited water supply, one respondent explained:

“We like to use expensive toilets which are durable but we cannot use them because water is limited, some of us are living where water is not close to our homes; we walk a long distance to get water.”

Also, the cost of constructing improved latrines was identified as another barrier. The average one-time cost of constructing a flush latrine with shelter which provides adequate privacy and protection was reported to be 1,303–2,170 USD, this cost was more than 100% of the average household monthly income in Babati. Moreover, the average cost of constructing a pit latrine with slab or VIP was reported to be 130–174 USD, which is also more than 100% of the average household monthly income in Babati. The cost of constructing a traditional pit latrine (without slab) was reported to be 8–22 USD, representing 7.9–22% of the average household monthly income in Babati. However, when the labour, including pit excavation, was provided by household members, this cost range further drops to the affordability threshold of 3–5%. One participant commented;

“I like improved latrine, but my financial situation is not good, I have to continue using the traditional one.”

The lack of adequate faecal sludge management services including sludge emptying services and treatment facilities in the town was also a deterrent for the households to construct improved and permanent sanitation facilities. Another barrier was the size of the land the household owned. Those with large pieces of land were able to construct new latrines when the ones they were using become full. One respondent argued:

“No one will empty a latrine while there is a big land around. When a latrine pit is full, that person will simply dig a new one.”

It was learned that emptying services were mainly provided by a municipal tanker for a charge of USD 30, which is about 29.7% of the monthly household income in Babati. This cost was considered by the majority of respondents as too expensive to afford. The willingness to pay exercise revealed that many respondents, especially those on the fringe areas of Babati town were not willing to change their common practice of digging a new pit when their old one was full.

With regards to sanitation status, the household survey and spot-check revealed that 3.3% of households had no sanitation facilities, hence presumed as practising open defecation, and 19.1% were using pit latrines without slabs (Table 5).

**Table 5:** Type of sanitation facilities.

Variable	Frequency	%
Flush/pour-flush toilet (to a septic tank or pit latrine)	137	28.2
Flush/pour-flush toilet (not to the pit latrine or septic tank)	18	4.2
Pit latrine with slab	202	41.6
Pit latrine without slab/open pit	93	19.1
Ventilated improved pit (VIP) latrine	20	4.1
No sanitation facility (Open defecation)	16	3.3

The sanitation facilities were further classified as per the Joint Monitoring Programme (JMP) [22]. When Babati’s sanitation status is compared with the country’s sanitation status using the data from JMP [23], the percentage of the safely managed sanitation services available in Babati is higher than the overall percentage for the national coverage percentages. On the other hand, the town has higher percentages of unimproved sanitation services and open defecation than the national coverage (Table 6).

**Table 6:** Sanitation classification as per JMP.

	Babati Status	National status (Urban) ^§^
	%	%
Safely managed sanitation services*	67.9	34.8
Basic sanitation services**	3.3	12.6
Limited sanitation services^	6.4	42.1
Unimproved sanitation services^^	19.1	9.2
Open defecation ^†^	3.3	1.4

*improved facilities that are not shared with other households and where excreta are safely disposed in situ or transported and treated off-site

**Use of improved facilities which are not shared with other households

^Use of improved facilities shared between two or more households

^^Use of pit latrines without a slab or platform, hanging latrines, or bucket latrines

^†^ Disposal of human faeces in fields, forests, bushes, open bodies of water, beaches, and other open spaces or with solid waste

^**§**^The 2020 Tanzania’s national data for sanitation from the JMP website [23]

The comparison of the relationships between various factors and a household latrine type showed an association between the type of the latrine the household owned and the level of education of the head of the household head (p = 0.003), household monthly income (p = 0.017), residence ownership (p = 0.001), wealth quintile (p = 0.007) and the source of water a household uses (p < 0.001) (Table 7).

**Table 7:** Chi-square results: Relationship between latrine type and household characteristics.

	Chi-square	df	p-value
Sex of the head of the household	1.95	4	0.745
The education level of the household head	31.93	8	0.003*
Marital status of the household head	9.26	12	0.68
Family size	9.22	8	0.324
Household monthly income	30.3	16	0.017*
Residence ownership	25.94	8	0.001*
Wealth Quintile	26.15	4	0.007*
Source of domestic water	74.09	16	0.001*

* = p<0.005

The multinomial logistic regression test indicated that five predictors: education level, household income, residence ownership, wealth quintile and source of domestic water explained 34.2% of the variability of the type of the latrine built by the household in the model (Nagelkerke R^2^ = 0.34). Significant coefficients were observed in education, wealth quantile, residence ownership and source of water. Pertaining to education, those with no formal education were more likely to construct pit latrines rather than improved latrine (flush/pour-flush toilet) connected to the septic tank (OR = 2.02, 95% CI 1.07–3.81, p = 0.031). The findings also show that those in the high wealth quintile were less likely to own flush/pour-flush toilet not connected to the septic tank (OR = 0.04, 95% CI .00–0.35, p = 0.004), which means they were more likely to own latrine connected to the septic tank. Privately owned residences were more likely to have pit latrines without a slab (OR = 15.47, 95%CI 1.91–125, p = 0.01) and pit latrine with a slab (OR = 2.49, 95%CI 1.14–5.47, p = 0.023) and less likely to construct flush or pour-flush toilets (connected to the septic tank). On the other hand, households that were collecting water from boreholes were more likely to own pit latrines without a slab (OR = 37.68, 95%CI 6.89–206.1, p<0.001) than households that were buying water from vendors (OR = 12.63, 95%CI 2.53–63.18, p = 0.002), households that were fetching from river/canal/spring/lake (OR = 19.26, 95%CI 3.97–93.34, p<0.001) and households that were getting water from other sources such as neighbours’ wells or neighbours’ tap water and rainwater (OR = 10.99, 95%CI 3.38–35.79, p<0.001).

Motives and barriers to practice improved sanitation behaviour in schools and college settings were largely dependent on the availability and use of sanitation facilities in schools. FGDs in schools and colleges revealed that most students were not satisfied with the sanitation situation in their schools. The main barrier for using latrines at school was the low drop-holes to student ratio. For the 33 schools and vocational training colleges surveyed, it was observed that the average ratio was 1:50 (drop-hole per male students) and 1:47 (drop-hole per female students). The highest number of students per drop holes observed was 1:400 (drop-holes per male students) and 1:198 (drop-holes per female students). Other barriers to practising improved sanitation behaviour in schools and college settings were limited availability of water in the latrines and lack of privacy as many school latrines had no doors. A spot-check exercise showed that more than 29% of the female latrines and 44% of the male latrines in schools and college settings were found to be dirty (presence of human excreta and/or worms could be seen on the floor/drop hole or a wall). Additionally, 41% of the female latrines and 47% of the male latrines had a very strong smell. The problem was more acute in public schools that have installed flush toilets because most of the flushing systems were not functioning. A student who wanted to use a latrine was required to carry a bucket of water for flushing. However, FGD with students revealed that most students were not observing that requirement, as a result, the latrines were often not flushed. Visible urine and faeces could be seen in the toilet pans during spot-check exercises. Most of the boys at the schools admitted to practise open defecation while they are at school, whilst girls reported to hold stool or urine until they get back home. Some students also reported that their parents have forbidden them to drink water while they are in school so that they can avoid using the school latrines to urinate.

### 4.3. Motives and barriers to invest in improved hygiene behaviour

#### 4.3.1. Handwashing with soap and water

FDGs and Motive and Barrier exercises revealed that the feeling of disgust was one of the main motives for washing hands. The respondents argued that one of the main reasons they wash hands was simply to remove dirt or dust from their hands. The second motive was comfort. Respondents said that they often wash their hands when they see a handwashing station close to the latrine or kitchen. They did not feel comfortable walking far from the latrine to wash their hands. The third motive was attraction, with respondents saying that they often wash their hands to look clean and walk around with clean hands. The last motive was fear of contracting diseases, where respondents claimed that they understand that eating with dirty hands can lead to contract diarrhoea or other stomach related diseases. Hence, they were impelled to wash hands to prevent themselves from contracting the diseases. However, they admitted to wash hands during diseases outbreaks, but were less likely when the outbreaks were contained. FGDs also showed that health information provided by the health officers in their communities encouraged them to construct handwashing stations and practice handwashing during this time.

On the other hand, financial constraints for buying soap or constructing handwashing facilities, a person’s attitude and religious beliefs such as the belief that human health is in God’s hands, were identified as major barriers to handwashing practice. However, the respondents who reported low income as one of the barriers to buy and use soap for handwashing in their households, admitted that they do buy soap for other domestic use such as laundry and bathing. So, it is the priority rather than availability what matters. Another key barrier reported was limited water availability. During FGDs, one respondent argued:

“Availability of water is a problem here…. when a place has no water, a person cannot wash hands properly”.

The household survey revealed that 17.3% of the households were not practising hand washing because the cost of buying soap was too high. However, 14% were using ash, mud and other materials as alternatives to soap. Access to a handwashing facility was also revealed to be a barrier to handwashing practices, as only 5.6% of households had handwashing stations. On the other hand, the comparison of the relationships between the presence of handwashing station and household characteristics showed that household demographic characteristics, education level, household monthly income and location of the source of domestic water are associated with the presence of a handwashing facility in the household (Table 8).

**Table 8:** Chi-Square results: Relationship between the presence of handwashing station and household characteristics.

Variable	Chi-Square	df	p-value
Sex of the head of the household	3.24	1	0.072
Education level	10.49	2	0.005*
Marital status	3.88	3	0.276
Family size	0.698	2	0.705
Household monthly income	14.87	4	0.005*
Residence ownership	0.23	2	0.892
Wealth quintile	2.45	1	0.118
Source of domestic water	16.29	4	0.003*

* = P<0.05

The binary logistic regression test indicated that three predictors; education level, household monthly income and source of domestic water explained about 14% of the variability of the handwashing station (Nagelkerke R^2^ = 0.138). Among the three predictors, only two: household monthly income and the source of domestic water were significant. This means, that although the Chi-Square tests showed significant association with the education level, there was no strong influence of this variable to predict/influence the presence of a handwashing station in a household. The results indicate that households that get water from other sources (neighbours’ wells or neighbour’s tap water, rainwater harvest, etc.) were less likely to have a handwashing station (OR = 0.429, 95%CI 0.18–1.01, p = 0.01). Also, as income increases, the likelihood of having a handwashing station in the household increases (OR = 1.002, 95%CI 1–1.003, p = 0.026).

In schools and colleges, the majority of the boys admitted that they do not wash their hands after coming from the latrine, especially after urination, however, that they would wash their hands before eating. During the FGD sessions, respondents argued that hands can rarely get contaminated when urinating hence there is no need to wash your hands afterwards. However, girls reported washing their hands after using the latrines and before eating but in all cases, soap was rarely used.

#### 4.3.2. Water treatment practices

FDGs revealed several motives and barriers for households to treat water. First, FGDs showed that fear of contracting water-borne diseases such as cholera and diarrhoea served as a motive for households to treat drinking water. Second, the type of water source used by the household, acted as both a barrier and motive for a household to treat drinking water. Households that were collecting water from sources that are considered to be contaminated such as a river or a lake where animals also drink from, felt the need to treat water before drinking. However, households that were collecting water from the town water supply authority source (piped water), did not feel the need to treat the water because the source was considered to be clean and safe. Third, the cost of treating water (financial constraints) was another barrier. Fuel cost i.e. cost of firewood, charcoal or gas used to boil water, and cost of buying water treatment chemicals impeded households to treat drinking water. One FGDs participant argued:

“Our income is still low, we can`t afford to buy fuel for boiling water.”

Fourth, belief in supernatural or divine powers influenced the household’s decision to treat water. There are common beliefs among some of the households that water from a spring is not contaminated, and God can protect people who drink water without treating it as long as people put their trust in him. Some respondents also argued that people treat water out of fear, and that fear makes them feel sick after drinking untreated water even when they are not sick. They argued, however, that those who are not afraid are less likely to get sick after drinking untreated water. One participant argued:

“If you believe from your heart that water is not contaminated, you will see that there is no reason to boil it, and if you drink it, you will not be sick… . it is only fear [that makes you feel sick].”

The fifth factor was family size. FGDs revealed that a large family size impeded households to treat drinking water and vice versa. This was also associated with cost as participants argued that it was difficult to treat water for everybody when the family is large. One FGD participant argued:

“Other households have large family sizes…there are so many family members….… it is difficult to boil water for ten people every day”

On the other hand, the household survey revealed that more than half (54.5%) of households do not treat water before drinking. It also shows that, of 45.5% who treat water, 37.4% boiled the water, and the rest use different methods such as filtration, chlorination and solar disinfection. There was also a significant association between water treatment practice and water source (p < 0.000). The study also observed the positive association between water treatment behaviour and household income (Table 9).

**Table 9:** Chi-Square results: Relationship between the water treatment and household characteristics.

Variable	Chi-Square	df	p-value
Sex of the Head of the household	0.067	1	0.796
Education level	5.14	2	0.077
Marital status	3.69	3	0.297
Family size	4.44	2	0.109
Household monthly income	9.164	4	0.050*
Residence ownership	3.273	2	0.195
Wealth quintile	0.72	1	0.788
Source of domestic water	17	4	0.001*

* = P <0.05

The results of the binary logistic regression test indicated that two predictors: water source and income level explained 6% of the variability of the water treatment (Nagelkerke R^2^ = 0.06). However, contrary to FGDs revelations, the test shows that households that get water from non-piped water sources (river and lakes) are less likely to treat drinking water (OR = 0.456, 95%CI 0.29–0.73, p = 0.001). However, as the household’s income increases, they become more likely to treat water before drinking (OR = 1.001, 95%CI 1–1.003, p = 0.049).

Pertaining to water treatment in schools, FGDs revealed that most of the students studying in public schools, drink water directly from the tap. Some of the students said that they would normally avoid drinking water while at the school, and would wait until they return home to where they could drink treated water. The situation was different in private schools where water was being treated by boiling or using water-guard or chlorine. Yet, students rarely use it. Most of them preferred tap water that they claimed had a better taste. On the other hand, college students purchased bottled water, not for health or safety concerns, but simply to avoid drinking salty tap water.

#### 4.3.3. Sanitation related hygiene behaviours: Use, cleanliness and child faeces disposal

FGDs unveiled four motives for cleaning latrines. First, social influence or the feeling of being embarrassed by neighbours. Second, fear of being fined by health officers, who occasionally inspects households and fine those whose latrines are found to be dirty. Third, the feeling of disgust when using a latrine with visible faeces and urine, and fourth, fear of contracting diseases from a dirty latrine. For barriers, FGDs revealed two key factors, namely poorly maintained floors and limited water availability. Most of the latrines had mud or wooden slabs which cannot be easily washed or cleaned using water. Households that had installed pour-flush or flush toilets also identified a lack of water as the main reason for having dirty latrines. FGD participants stated fear of contracting urinary tract infections (UTIs) and fungal diseases while using flush toilets when water is limited. A participant in one of FGDs argued:

“(Pour) flush toilets are the ones causing UTI to people, this is because, some people go there and they do not pour water”.

Limited availability of water to the places where flush toilets have been installed was argued to influence some adult individuals to defecate outside, in an attempt to avoid the use of dirty or un-flushed toilets. The findings revealed that the majority of the households with children under five years of age were not following proper child faeces disposal practices. It was learned that 55% of these households were disposing child faeces in the toilets and the rest were disposing elsewhere. The households that were not disposing child faeces in the toilet, reported that they felt a child’s faeces are less harmful.

#### 4.3.4 Menstrual hygiene management

The major motives for practising proper menstrual hygiene (e.g. use of clean pads) included feeling confident, comfortable and fear of leaking menstrual blood. Whereas, barriers to maintain proper menstrual hygiene were limited availability of sanitary pads, cost and lack of designated places to dispose of used sanitary materials. FGDs revealed that the majority of women, especially in rural areas, were using pieces of cloths during menstruation to protect themselves. Only a few respondents reported using ready-made sanitary pads. A participant in one of the women FGDs stated that:

“Our fellows living in town use pieces of cotton or sanitary pads…for us, buying cotton or sanitary pads is not often possible because they are expensive, we use pieces of clothes”.

The common practice for disposing of sanitary pads is through burning or disposing in the pit latrine or disposing with other domestic wastes. During the FGD, one of the participants said:

“For those using pads like me, after use, we normally burn them or dump them together with other domestic wastes waiting to be carried away by waste truck…. However, for those who are using pit latrines, they just throw the pads in the pit, unfortunately, there are some women who throw pads carelessly in the street”.

However, the use of sanitary pads is not well perceived by all women. Some complained that they do experience some kind of allergic reactions or infections after using sanitary pads they buy from the shops. One FGDs participant argued that:

“These sanitary pads indeed have negative effects…. Let’s speak the truth; some of my daughters have used them and experienced fungal infections which myself I had never suffered from, so it is better to use pieces of cloths”.

In schools, major barriers for practising proper menstrual hygiene were reported to be lack of sanitary pads, lack of changing rooms in schools, and limited availability of water and soap in the school latrines. FGDs in primary and secondary schools revealed that students were missing classes because of a lack of sanitary pads. The following were some of the comments from two girls during FGD in schools;

“Yes, sometimes I miss school as I fear……. I may wet myself, and if that occurs my fellow students will laugh at me”.

“When I request money for buying pads, sometimes my mother tells me that she doesn’t have money, I just stay at home until the business ends because I find it difficult to use pieces of cloth when I am in school”.

Additionally, the absence of a place to dispose of sanitary pads hindered school girls to practice proper menstrual hygiene behaviour. The lack of containers to dispose of sanitary pads impelled school girls to dispose of them in the school drop holes, leading to clogged latrines. During the FGDs girls admitted that they prefer using pit latrines in schools because they can easily dispose used sanitary pads in the pit latrines compared to flush toilets.

## 5. Discussion

### 5.1 Motives and barriers to invest in sanitation

Babati Town, like many urban areas in Tanzania and the developing world, has a rapidly growing human population. However, the growth in urban populations is not happening hand in hand with growth in the provision of sanitation and hygiene services. At the household and individual level, investment in improved sanitation facilities and hygiene behaviour is affected by many factors. The key motive for a household to construct a latrine is the fear of being punished by town authorities. This implies that, having clear rules, regulations and guidelines on the construction of latrines, including standards, and strict enforcement of the same can improve sanitation condition in urban areas. Comfort also drives people’s desire to construct an improved latrine especially when people consider a latrine as a safer and more comfortable place to defecate than in the bushes or other places outside. The need to impress neighbours and friends was found to be vital in bringing positive change through social pressure. On the other hand, children safety (nurture) plays a role in influencing a household to construct an improved latrine.

On barriers, a household’s economic situation and the cost of constructing improved latrines prevent households from adopting improved latrines. The costs of constructing flush latrines, pit latrines with slab and VIP latrines with proper shelter are exceeding the affordability threshold of 3–5% which is an international benchmark for water and sanitation expenditure as a percentage of household income [24, 25]. These factors were also observed to impede households from adopting improved sanitation facilities in Dar es Salaam [5]. However, the costs are largely a matter of perception and lack of awareness about available alternative materials and technologies for latrine construction. Several NGOs and private actors have developed and introduced into the market relatively cheap technologies for latrine construction, which would cost a household less than 3% of the average household income, making sanitation services more affordable to many households. Though these technologies have been featured in many exhibitions, information has not reached a wide number of households and they refer to traditionally known materials. This situation implies that there is a need for strategic efforts by both state and non-state actors to promote the adoption of the new toilet technologies made by locally available materials which are cheaper than imported materials. This can include awareness campaigns, development of guidelines and standards for the construction of latrines in urban areas, and mason training. On the other hand, environmental factors such as poor soil condition and a high-water table also impede households to invest in improved sanitation. Poor soil condition may cause latrine to collapse, and high-water table leads to the need to frequently empty the latrines. Poor soil condition problems might be solved by lining a pit latrine. Pit latrine lining is the process of constructing a pit latrine’s wall using brick, rot-resistant timber, concrete, stones, or mortar plastered onto the soil to make it stable. Although the use of these materials can also solve safety issues, it is associated with high costs inciting a vicious cycle on the use of unimproved latrines. Other types of latrines that will be suitable for such environmental conditions are above-ground designs such as the double vault VIP pit latrines, raised pit latrines, and anaerobic composting latrines as described by Harvey et al [26]. The double vault VIP pit latrine is the latrine with two shallow vaults that are built side by side. One vault is used initially then sealed when full. The second vault is then used until that is full, at which point the first vault can be emptied and reused. The raised pit latrine is the latrine whose pit is built upwards above the ground level. The anaerobic composting latrine is the toilet that uses a dry disposal system in which urine and faeces are managed separately.

The size of the land owned is another barrier for households to construct improved latrines, especially, in fringe areas of the town. A household that owns a large piece of land can easily dig a new pit if the one in use is full, hence avoiding emptying costs. Therefore, most do not see the need to invest in durable (permanent) and improved latrines.

Demographic and household characteristics were seen to influence a household’s decision to invest in improved latrines. Education level, property rights and a household’s source of water have a significant influence on a household’s decision to invest in improved latrines. On the other hand, education level influences household income, where households with household heads with secondary level of education or above are likely to have higher income and are more likely to construct an improved latrine. These findings are in line with a study by Mwakitalima et al [5], conducted in rural Tanzania, which asserts that households, where the household head had a secondary level of education or above, were more likely to have improved types of latrines than their counterparts.

The findings also show that privately owned houses were more likely to have pit latrines relatively, whereas those constructed specifically for renting were likely to have flush or pour-flush toilets. This may be because, most rental dwellings are found in the central business district (CBD), and most people who rent dwellings in the CBD are more likely to have higher education level or income, hence would demand better sanitation facilities. Most of the rental dwellings in the CBD are connected to the piped water supplied by the urban water supply authority, which is also a push factor for households to invest in improved latrines. These findings are, however, contrary to a study conducted in Ghana [27] and Uganda [28] which found that most of the rental houses had poor sanitation facilities because homeowners were less likely to invest in improved sanitation facilities for their tenants, and tenants were not willing to make sanitation investments in rental properties. However, this contradiction could be explained by fact that Babati is a small town, which is less than 15 years old since it was promoted to town status, hence most people who rent households are likely to be newcomers with a higher level of education or good income. On the other hand, the studies in Ghana and Uganda involved big and old cities and focused on unplanned areas where mostly lower-income families and people with lower education levels reside. The areas were poorly planned and the houses fetched low renting prices, hence homeowners had no incentive to invest in improved sanitation.

Households that collect water from sources other than piped water systems are less likely to own a flush or pour-flush toilet (connected to the septic tank) because a flush toilet would mean an increase in water costs or time to fetch water or both. Advanced statistical tests (multinomial logic regression analysis) have revealed that household income influence on the type of the latrine a household owned was insignificant, yet it is a key factor in a household decision to adopt an improved latrine because income has a direct influence on other significant factors such as water availability and accessibility (source of water), and property rights. Income is also associated with a low level of education, as in most cases those with a low level of education often end up having access to low-skilled and hence low paying jobs [29].

The sanitation situation in school settings is not satisfactory. Most of the school toilets were dirty, had a strong smell and had no running water, a situation that can lead to students avoiding using them. The average ratio of students to drop hole is below the Tanzanian minimum standards of students to drop hole ratio which is one toilet per 20 male students and one toilet per 20 female students [30, 31]. This situation is common in many schools in Tanzania. The 2018 WASH assessment in schools found that only 28% of schools in Tanzania met the minimum standard for the number of pupils per drop hole set by the Government [31].

### 5.2 Handwashing practices

The fear of contracting diseases motivated people to practice handwashing, however, this is mostly done when there are disease outbreaks in town. Previous studies also identified fear as one of the key motives for uptalking WASH behaviour [9, 32, 33]. However, previous evidence shows that fear is a temporary stimulus to change behaviours as when fear disappears so does the behaviours too [34]. The feeling of disgust due to the presence of dirt/dust in hands, bad smell in the latrine, protecting children and their future by washing hands (nurture) and the wish to look smart and clean also motivate individuals to wash hands. These emotional push factors can also be exploited in promotional campaigns to promote handwashing practices. Previous studies have shown that emotional push factors as nurture, disgust, affiliation and social status, and visual clues and nudges in behavioural places have a positive impact in changing key hygiene behaviours such as handwashing [35] and food hygiene [36]. The studies also show that health information provided by health workers and availability of facilities/materials can encourage handwashing practices. Availability of handwashing facilities and materials such as handwashing stations and soap were also found to encourage handwashing behaviour in schools in Myanmar [37] and Health Care Facilities in rural Tanzania [38]. In Bangladesh, studies found that having water and soap available at the place to wash hands after toileting with proper cues and nudges influenced hand washing after faecal contact [39]. This could explain why in both households and schools, although individuals claimed to wash hands after using latrines, also admitted to rarely use soap. The findings are also similar to what was reported by Thomas et al. [40] that although hand washing is widely practised in Tanzania, the use of soap is not common even in critical times such during disease outbreaks.

The level of education, water source and household income were also found to be associated with hand washing and water treatment practices. Households headed by individuals who have a secondary level of education or above are more likely to have handwashing stations compared to those with lower levels of education. This might be because household heads with a relatively high level of education are more likely to have knowledge of the needs for hygiene practices and hence install handwashing stations in their dwellings. On the other hand, dwellings that are not connected to the piped water system are less likely to own handwashing stations because of the associated nuisance of fetching water. Additionally, household income influences the presence of handwashing station in a dwelling, largely because of the costs associated with the installation of handwashing stations and the influence of income in access to piped water. This finding is consistent with Luby et al. [39] findings which reveal that the majority of dwellings connected to the piped water systems in rural Bangladesh were from higher socio-economic strata and likely to have more knowledge of water quality and safety, health issues and, therefore, likely to practice good hygiene behaviours; more so than families from lower socio-economic conditions.

### 5.3 Water treatment practices

Fear of contracting water-borne diseases especially cholera and diarrhoea, and household water source is found to influence a household’s decision to treat water but we know this might be temporary stimuli. These two push factors are linked as the use of contaminated water may cause an individual to contract diseases, leading to loss of income or loss of loved ones or both. Household members largely perceived water sourced from lakes and rivers to be contaminated because these sources are open, and are sometimes used by livestock and wild animals. Financial constraint also emerges as a significant deterrent in water treatment practices because the common method for water treatment is boiling. This means a household has to invest in fuel for boiling water, which translates to financial challenge as in many parts of the country; cooking fuel is increasingly becoming too expensive or not readily available, especially gas and electricity.

### 5.4 Other sanitation related hygiene behaviours and menstrual hygiene management

Although the fear of contracting diseases, protecting their children (nurture) and feeling of disgust for using a dirty latrine emerged among key motivating factors for practising improved hygiene behaviour, most households cleaned their latrines to avoid fines and to impress neighbours and visitors. This means social status or social influence and fear of being fined by town authorities play a major role in influencing households to clean their latrines. However, the willingness to clean the latrines is hindered by poor latrines floor conditions as most do not have concrete. Houses and schools using flush toilets also face frequent water shortages or are not connected to the piped water, leading to poor cleaning conditions. The problem is more severe in public schools where water availability is a huge problem, impelling pupils to defecate in open areas in an attempt to avoid uncleaned or unflushed latrines, in fear of contracting diseases such as UTI, diarrhoea or chlorella. A study by Garn et al. [41] also found that a dirty facility was more likely to deter students especially girls from using them. Pertaining to the proper disposal of child faeces, many people still do not regard child faces as harmful, hence do not see the need to properly dispose of them. Unsafe disposal of child faeces was also reported in other studies, arguably because child faeces is believed to not be harmful [42, 43]. The poorly conceived safety may be due to the innocence of children, therefore promotional campaigns to change this belief must be designed in such a way that caretakers would continue to care for the children with passion and love, and without the feeling of disgust about the children needing help to be cleaned.

Empirical evidence in this study shows that the presence of changing rooms for girls in schools as well as the availability of sanitary pads motivates girls to practice good menstrual hygiene. Yet, in Babati town, menstrual hygiene management is a neglected issue as little attention has been given to this subject. Schools do not have changing rooms, and most of the women living in a fringe part of the town and school girls do not have access to sanitary products or are unable to afford such products. They largely rely on reusable and washable pieces of cloth. Some women use non-absorbent and uncomfortable menstrual cloths. The challenges in limited access to sanitary products and materials were also reported in other studies [44–46]. The argument that the use of sanitary pads causes allergic reactions or lead to urinary tract infections might be the result of wearing sanitary pads for a long time without changing to reduce the costs or because of lack of access to changing rooms. According to Sommer et al. [17], the problem of poor menstrual management among school girls due to the limited availability of facilities and materials is common in low- and middle-income countries. Chinyama [47] also found inadequate provision of sanitary materials, water and sanitation facilities in schools in Zambia. The challenges including a lack of facilities and material for young girls often compromise their school attendance and participation in physical activities when menstruating due to fear of being teased and embarrassed by menstrual leakage [47, 48]. Lack of bins to dispose of sanitary resulted in school girls disposing of the pads in flush toilets, clogging the flushing system in schools. This situation negatively affects sanitation facilities in schools, increasing the potential for disease outbreaks.

Our study had several limitations. First, we did not collect direct observation data of students’ behaviour. The direct observation could provide additional insight on the actual behaviour and barriers in school settings. Second, we did not organize specific FGD session for people with disability. Thus, data on the sanitation and hygiene barriers facing those with disabilities were not adequately collected. Third, in some wards, the enumerators had to be escorted by the wards executive officers (WEOs) who helped them to identify the sampled households. WEOs were sometimes working as sanitation and hygiene inspection officers. To avoid the chances of WEOs influencing responses, enumerators explained to the respondents that the officials were not part of the research team. Moreover, the respondents were assured confidentiality and anonymity.

## 6. Conclusion

This study has identified structural, social and economic motives and barriers that influence households to invest in improved sanitation facilities and practice safe sanitation behaviour. It shows that both motives and barriers are interconnected and multi-layered. This is a call for the need to address them in totality rather than individually. There is a need to set out a mechanism for enforcement as evidence shows that the fear of being fined pushes people to adopt improved sanitation and hygiene facilities. Socially, it is important to recognise the taboos and traditions that impeded improvement and use socially available mechanisms to bring change. This could include the use of local leaders and elders to advocate for proper sanitation and hygiene practices. Lastly, from an economic view, there should be efforts to address existing financial constraints to meet the needs for sanitation and water for households with lower incomes. Provision of sanitation loans is one of the viable options to consider. The sanitation loans have been proved to help low-income households to install, upgrade or repair latrines by Sijbesma et al [49]. The government in the countries of the global south need to understand health benefit translates to economic growth and national prosperity. Thus, they need to provide incentives such as tax breaks and subsidies to sanitation and hygiene materials. This study provides empirical evidence on what drives or blocks households from investing in improved sanitation and hygiene to policymakers and public health practitioners who design and execute sanitation and hygiene intervention programs. While many programs focus on health reasons, this study revealed that in many cases social status or social influence, disgust, nurture, comfort and fear of being fined by town authorities play a major role in influencing households to practice improved sanitation and hygiene behaviour. However, this study focused on one growing small town in Tanzania. To gain a more complete picture of sanitation challenges, this study should be replicated in other small towns of Tanzania.

## Supporting information

S1 Table Multinomial logistic regression test results—Household latrine type vs level of education, household monthly income, residence ownership, wealth quintile and the source of water. (DOCX)

S2 Table Binary logistic regression test results—Presence of hand wash station vs education level, income and source of domestic water. (DOCX)

S3 Table Binary logistic regression test results—Household water treatment practices vs source of domestic water and household income. (DOCX)
