# Supplementary material for: What influences individuals to invest in improved sanitation services and hygiene behaviours in a small town? A formative research study in Babati, Tanzania
Source: PLoS One. 2022 Jul 21;17(7):e0270688. doi: 10.1371/journal.pone.0270688 (PMC9302730; doi:10.1371/journal.pone.0270688)
Supplement: S1 Table — (DOCX) [file pone.0270688.s001.docx]

**S1 Table:** Multinomial logistic regression test results - Household latrine type vs level of education, household monthly income, residence ownership, wealth quintile and the source of water

| **TYPE OF**  **LATRINE^a^** |  | **B** | **Wald** | **df** | **Sig.** | **Exp(B)** | **95% CI for Exp(B)** |
| --- | --- | --- | --- | --- | --- | --- | --- |
| Flush/pour flush (not to pit latrine or septic tank) | Intercept | 1.892 | 1.803 | 1 | 0.179 |  |  |
|  | Income | -0.001 | 0.338 | 1 | 0.561 | 0.999 | (0.995, 1.003) |
|  | Privately owned house | -0.301 | 0.148 | 1 | 0.701 | 0.74 | (0.159, 3.44) |
|  | Relative house | 1.093 | 0.612 | 1 | 0.434 | 2.983 | (0.193, 46.129) |
|  | Rental house | 0^b^ | . | 0 | . | . | . |
|  | No formal education | -0.02 | 0 | 1 | 0.990 | 0.98 | (0.047, 20.249) |
|  | Primary education | -0.944 | 1.587 | 1 | 0.208 | 0.389 | (0.09, 1.69) |
|  | Secondary education & above | 0^b^ | . | 0 | . | . | . |
|  | High | -3.299 | 8.343 | 1 | 0.004* | 0.037 | (0.004, 0.346) |
|  | Second‎‎/Third‎/Fourth | 0^b^ | . | 0 | . | . | . |
|  | Borehole | -16.422 | 0 | 1 | 0.998 | 7.38E-08 | (0, c) |
|  | Vendors | -17.123 | 0 | 1 | 0.997 | 3.66E-08 | (0, c) |
|  | River/Canal/Spring/lake | -0.347 | 0.063 | 1 | 0.802 | 0.707 | (0.047, 10571) |
|  | Others | -0.442 | 0.3 | 1 | 0.584 | 0.643 | (0.132, 3.125) |
|  | Connected to water supply | 0^b^ | . | 0 | . | . | . |
| Pit latrine without slab/open pit | Intercept | -4.263 | 7.432 | 1 | 0.006 |  |  |
|  | Income | -0.002 | 1.862 | 1 | 0.172 | 0.998 | (0.994, 1.001) |
|  | Privately owned house | 2.739 | 6.601 | 1 | 0.010* | 15.471 | (1.915, 125.008) |
|  | Relative house | -14.749 | 0 | 1 | 0.997 | 3.93E-07 | (0,c) |
|  | Rental house | 0^b^ | . | 0 | . | . | . |
|  | No formal education | 1.828 | 2.922 | 1 | 0.087 | 6.223 | (0.765, 50.625) |
|  | Primary education | 0.898 | 2.027 | 1 | 0.154 | 2.455 | (0.713, 8.452) |
|  | Secondary education & above | 0^b^ | . | 0 | . | . | . |
|  | High | -1.369 | 2.644 | 1 | 0.104 | 0.254 | (0.049, 1.324) |
|  | Second‎‎/Third‎/Fourth | 0^b^ | . | 0 | . | . | . |
|  | Borehole | 3.629 | 17.521 | 1 | 0.000* | 37.678 | (6.888, 206.099) |
|  | Vendors | 2.536 | 9.54 | 1 | 0.002* | 12.634 | (2.527, 63.176) |
|  | River/Canal/Spring/lake | 2.958 | 13.49 | 1 | 0.000* | 19.257 | (3.973, 93.343) |
|  | Others | 2.397 | 15.845 | 1 | 0.000* | 10.994 | (3.377, 35.794) |
|  | Connected to water supply | 0^b^ | . | 0 | . | . | . |
| Pit latrine with slab | Intercept | -0.019 | 0 | 1 | 0.984 |  |  |
|  | Income | -0.001 | 1.198 | 1 | 0.274 | 0.999 | (0.998, 1.001) |
|  | Privately owned house | 0.913 | 5.18 | 1 | 0.023* | 2.493 | (1.135, 5.473) |
|  | Relative house | 0.817 | 0.839 | 1 | 0.36 | 2.263 | (0.394, 12.986) |
|  | Rental house | 0^b^ | . | 0 | . | . | . |
|  | No formal education | 2.055 | 5.962 | 1 | 0.015* | 7.807 | (1.5, 40.63) |
|  | Primary education | 0.556 | 2.111 | 1 | 0.146 | 1.743 | (0.824, 3.689) |
|  | Secondary education & above | 0^b^ | . | 0 | . | . | . |
|  | High | -1.387 | 3.043 | 1 | 0.081 | 0.25 | (0.053, 1.187) |
|  | Second‎‎/Third‎/Fourth | 0^b^ | . | 0 | . | . | . |
|  | Borehole | 0.955 | 1.73 | 1 | 0.188 | 2.6 | (0.626, 10.796) |
|  | Vendors | 0.306 | 0.231 | 1 | 0.631 | 1.358 | (0.389, 4.737) |
|  | River/Canal/Spring/lake | 0.918 | 2.113 | 1 | 0.146 | 2.503 | (0.726, 8628) |
|  | Others | 0.701 | 4.679 | 1 | 0.031* | 2.016 | (1.068, 3.806) |
|  | Connected to water supply | 0^b^ | . | 0 | . | . | . |
| Ventilated improved pit (VIP) latrine | Intercept | -18.894 | 367.904 | 1 | 0 |  |  |
|  | Income | 0 | 0.001 | 1 | 0.974 | 1 | (0.998, 1.002) |
|  | Privately owned house | 0.308 | 0.186 | 1 | 0.667 | 1.361 | (0.335, 5.526) |
|  | Relative house | 1.285 | 0.887 | 1 | 0.346 | 3.616 | (0.249, 52.481) |
|  | Rental house | 0^b^ | . | 0 | . | . | . |
|  | No formal education | 1.805 | 1.552 | 1 | 0.213 | 6.08 | (0.355, 104.031) |
|  | Primary education | 0.621 | 0.569 | 1 | 0.451 | 1.861 | (0.371, 9.348) |
|  | Secondary education & above | 0^b^ | . | 0 | . | . | . |
|  | High | 15.816 | . | 1 | . | 7.40E+06 | (7.40E+06, 7.40E+06) |
|  | Second‎‎/Third‎/Fourth | 0^b^ | . | 0 | . | . | . |
|  | Borehole | -16.419 | 0 | 1 | 0.998 | 7.40E-08 | (0,c) |
|  | Vendors | 0.548 | 0.21 | 1 | 0.647 | 1.73 | (0.166, 18.076) |
|  | River/Canal/Spring/lake | -15.689 | 0 | 1 | 0.997 | 1.54E-07 | (0,c) |
|  | Others | 0.984 | 2.633 | 1 | 0.105 | 2.674 | (0.815, 8.774) |
|  | Connected to water supply | 0^b^ | . | 0 | . | . | . |
| a. The reference category is: Flush or pour-flush (to septic tank or pit latrine).  b. This parameter is set to zero because it is redundant.  c. Floating point overflow occurred while computing this statistic. Its value is therefore set to system missing.  **= Significant values (P values <0.05)* | | | | | | | |
