# Supplementary material for: What influences individuals to invest in improved sanitation services and hygiene behaviours in a small town? A formative research study in Babati, Tanzania
Source: PLoS One. 2022 Jul 21;17(7):e0270688. doi: 10.1371/journal.pone.0270688 (PMC9302730; doi:10.1371/journal.pone.0270688)
Supplement: S2 Table — (DOCX) [file pone.0270688.s002.docx]

**S2 Table:** Binary logistic regression test results **-** Presence of hand wash station Vs education level, income and source of domestic water

|  | **B** | **Wald** | **df** | **Sig.** | **Exp(B)** | **95% C.I. for EXP(B)** |
| --- | --- | --- | --- | --- | --- | --- |
| Income | .002 | 4.949 | 1 | 0.026* | 1.002 | (1.000, 1.003) |
| Secondary education &above (reference) |  | 1.649 | 2 | 0.438 |  |  |
| *No formal education* | -.794 | .883 | 1 | 0.347 | .452 | (0.086, 2.367) |
| *Primary education* | -.524 | 1.383 | 1 | 0.240 | .592 | (0.248, 1.418) |
| Household is connected (reference) |  | 4.750 | 4 | 0.314 |  |  |
| *Borehole* | -19.407 | .000 | 1 | 0.998 | .000 | 0.000 |
| *Buy from vendors* | -1.280 | 1.460 | 1 | 0.227 | .278 | (0.035, 2.217) |
| *River/Spring/Canal/lake* | -.543 | .663 | 1 | 0.415 | .581 | (0.157, 2.146) |
| *From other sources* | -.845 | 3.765 | 1 | 0.010 | .429 | (0.183, 1.009) |
| Constant | -1.395 | 11.416 | 1 | 0.001 | .248 |  |

**= Significant values (P<0.05)*
