# Supplementary material for: What influences individuals to invest in improved sanitation services and hygiene behaviours in a small town? A formative research study in Babati, Tanzania
Source: PLoS One. 2022 Jul 21;17(7):e0270688. doi: 10.1371/journal.pone.0270688 (PMC9302730; doi:10.1371/journal.pone.0270688)
Supplement: S3 Table — (DOCX) [file pone.0270688.s003.docx]

**S3 Table:** Binary logistic regression test results - Household water treatment practices vs Source of domestic water and Household income

|  | **B** | **Wald** | **df** | **Sig.** | **Exp(B)** | **95% C.I. for EXP(B)** |
| --- | --- | --- | --- | --- | --- | --- |
| Income | .001 | 3.875 | 1 | 0.049* | 1.001 | (1.000, 1.003) |
| HH is connected (*Reference*) |  | 11.156 | 4 | 0.025 |  |  |
| *Borehole* | -.308 | .543 | 1 | 0.461 | .735 | (0.324, 1.666) |
| *Buy from vendors* | -.560 | 1.619 | 1 | 0.203 | .571 | (0.241, 1.353) |
| *River/Spring/Canal/lake* | -.306 | .738 | 1 | 0.390 | .737 | (0.367, 1.479) |
| *From other sources* | -.786 | 10.817 | 1 | 0.001* | .456 | (0.285, 0.728) |
| Constant | .203 | 1.105 | 1 | 0.293 | 1.225 |  |

**= Significant values (P<0.05)*
